# Combined transcutaneous electrical nerve stimulation and low-level laser photobiomodulation for postoperative pain after cesarean section: A randomized controlled trial

**DOI:** 10.1371/journal.pone.0360761

**Published:** 2026-10-22

**Authors:** Alane Macatrão Pires de Holanda Araújo Sena, Ivan Daniel Bezerra Nogueira, Kássio Rafael Rocha de Sena, Íllia Nadinne Dantas Florentino Lima, Patrícia Angélica de Miranda Silva Nogueira

**Affiliations:** 1 Postgraduate Program in Physiotherapy (PPGFIS), Federal University of Rio Grande do Norte (UFRN), Natal, Rio Grande do Norte, Brazil; 2 Department of Physical Therapy, Federal University of Rio Grande do Norte (UFRN), Natal, Rio Grande do Norte, Brazil; 3 Faculdade Estácio de Natal, Natal, Rio Grande do Norte, Brazil; Massachusetts General Hospital, UNITED STATES OF AMERICA

## Abstract

**Introduction:**

Postoperative pain after cesarean section may delay recovery and impair function. Transcutaneous electrical nerve stimulation (TENS) and low-level laser photobiomodulation (PBM) are used for pain control, but the additional effect of combining these therapies remains unclear. This study investigated this effect.

**Methods:**

A randomized clinical trial with four groups: combined TENS + low-level laser photobiomodulation (PBM), TENS alone, placebo, and control. Assessments were performed at 8–12 h (t1), 20–24 h (t2), and 44–48 h postpartum (t3). Outcomes included pain intensity, functional independence, functional capacity assessed by the Two-Minute Walk Test (2MWT), and cardiorespiratory variables. Two intervention sessions were applied after t1 and t2, except in the control group. Data were analyzed using mixed ANOVA and nonparametric equivalents when appropriate. Statistical significance was set at p < 0.05.

**Results:**

A total of 88 women were included. Pain intensity decreased significantly over time in the combined TENS + PBM group (1.82 ± 1.18 at t2 vs 1.30 ± 0.86 at t3; p = 0.008) and in the TENS group (1.59 ± 1.05 at t2 vs 0.95 ± 1.05 at t3; p = 0.025). Functional independence increased in the combined therapy group (14.9 ± 3.3 at t1 vs 17.1 ± 3.6 at t3; p = 0.034) and in the TENS group (15.2 ± 3.0 at t1 vs 17.5 ± 3.4 at t3; p = 0.028). Functional capacity improved in all groups between t2 and t3 in the Two-Minute Walk Test (Control: 63.5 vs 76.8 m, p = 0.002; Placebo: 50.3 vs 63.9 m, p = 0.002; Combined: 48.3 vs 65.2 m, p = 0.0006; TENS: 61.1 vs 72.2 m, p = 0.005). No significant differences were observed between groups (p > 0.05). A significant time effect was found for pain (p < 0.05) and functional independence (p < 0.001).

**Conclusion:**

Pain decreased and functional independence improved during the postoperative period. However, the combination of TENS and low-level laser photobiomodulation did not provide additional benefits compared with the other groups.

## Introduction

Postoperative pain after cesarean section is typically characterized as nociceptive pain resulting from surgical tissue injury and the inflammatory phase of wound healing. This pain, usually located in the abdominal region, may interfere with maternal mobility and daily activities during the early postpartum period, including breastfeeding positioning, newborn care, sitting, standing, walking, and personal hygiene, particularly within the first 24 hours after delivery [1–5].

Pain perception involves complex mechanisms in which pain threshold and pain tolerance represent distinct components of nociception. Pain threshold refers to the point at which a stimulus is first perceived as painful and reflects the physiological sensitivity of nociceptors and the activation of afferent pathways. In contrast, pain tolerance represents the maximum intensity of pain that an individual can endure and is strongly influenced by emotional, cognitive, and contextual factors such as anxiety, attention, and previous experiences. While pain threshold is primarily associated with peripheral sensory processes, pain tolerance depends largely on central pain modulation mechanisms involving cortical and limbic system pathways [6].

Non-pharmacological strategies have been increasingly explored as adjunct approaches for postoperative pain management after cesarean delivery. Among these, low-level laser photobiomodulation has gained attention as a noninvasive and painless therapeutic modality capable of modulating inflammatory responses and promoting tissue repair. Photobiomodulation acts through the absorption of light photons by cellular chromophores, particularly cytochrome c oxidase in mitochondria, leading to increased adenosine triphosphate (ATP) production and activation of intracellular signaling pathways. These mechanisms may result in several physiological effects, including modulation of inflammatory mediators, stimulation of angiogenesis, and increased fibroblast activity, which contribute to tissue regeneration and pain reduction in surgical wounds [7–9].

Another widely used modality for pain control is transcutaneous electrical nerve stimulation (TENS), a noninvasive electrotherapeutic technique that delivers electrical currents through surface electrodes placed on the skin. TENS is believed to modulate pain through mechanisms such as activation of the gate control theory and stimulation of endogenous opioid pathways, leading to the release of analgesic neurotransmitters including endorphins [1,10]. Previous studies suggest that TENS may reduce postoperative pain and facilitate functional recovery in women after cesarean section, and it is generally well tolerated and feasible for use in hospital settings [11].

Although both photobiomodulation and TENS have shown potential benefits when applied individually, evidence regarding their combined use in postoperative cesarean wounds remains limited. The simultaneous application of these modalities may enhance analgesic effects and contribute to improved functional recovery during the early postpartum period [12].

Therefore, this study hypothesized that the combined use of TENS and low-level laser photobiomodulation would provide additional analgesic effects and promote improvements in functional capacity, functional independence, and physiological cardiorespiratory responses in women during the postoperative period after cesarean section. The aim of this study was to analyze the additional effect of the association between TENS and low-level laser photobiomodulation on postoperative pain in women undergoing cesarean delivery.

## Methods

### Study design and participants

This was a randomized, controlled, assessor-blinded clinical trial conducted in a maternity hospital in Northeast Brazil between July 2023 and June 2025. In the same period, the participants were followed and recruited through convenience sampling upon hospital admission for cesarean delivery (elective or emergency). Ethical approval was obtained from the Research Ethics Committee in March 2020. All data are in the manuscript and/or supporting information files.

Eligible participants were women within 8–12 hours post-cesarean section, clinically stable, without obstetric complications or active infections, and receiving standardized pharmacological management (anti-inflammatory, analgesic, and antiflatulent medications with controlled administration intervals). Exclusion criteria included: (1) prior use of transcutaneous electrical nerve stimulation (TENS); (2) presence of neurological or musculoskeletal disorders; (3) inability to complete the intervention protocol or the 2-minute walk test (2MWT); and (4) clinical or obstetric instability during the study period. Participants were randomly allocated into four groups: Control, Placebo, TENS alone, and TENS combined with low-level laser photobiomodulation (PBM). The study followed CONSORT guidelines.

### Randomization and blinding

Participants were randomly assigned to one of the four groups using a computer-generated randomization sequence. Allocation concealment was ensured using sealed, opaque envelopes prepared by an independent researcher. Outcome assessors and data analysts were blinded to group allocation. Due to the nature of the interventions, therapists were not blinded. Participants in the placebo group received simulated interventions to maintain blinding.

### Intervention protocol

Participants underwent two intervention sessions according to group allocation, except for the Control group, which received no intervention. The first session was conducted 8–12 hours after cesarean delivery (t1), and the second session at 20–24 hours (t2). At both t1 and t2, the interventions were performed after the participants’ assessments. Follow-up assessments were performed at 44–48 hours postpartum (t3). The participants were follow-up between 8–12 hours and 44–48 hours after cesarean. (Fig 1)

**Fig 1:**
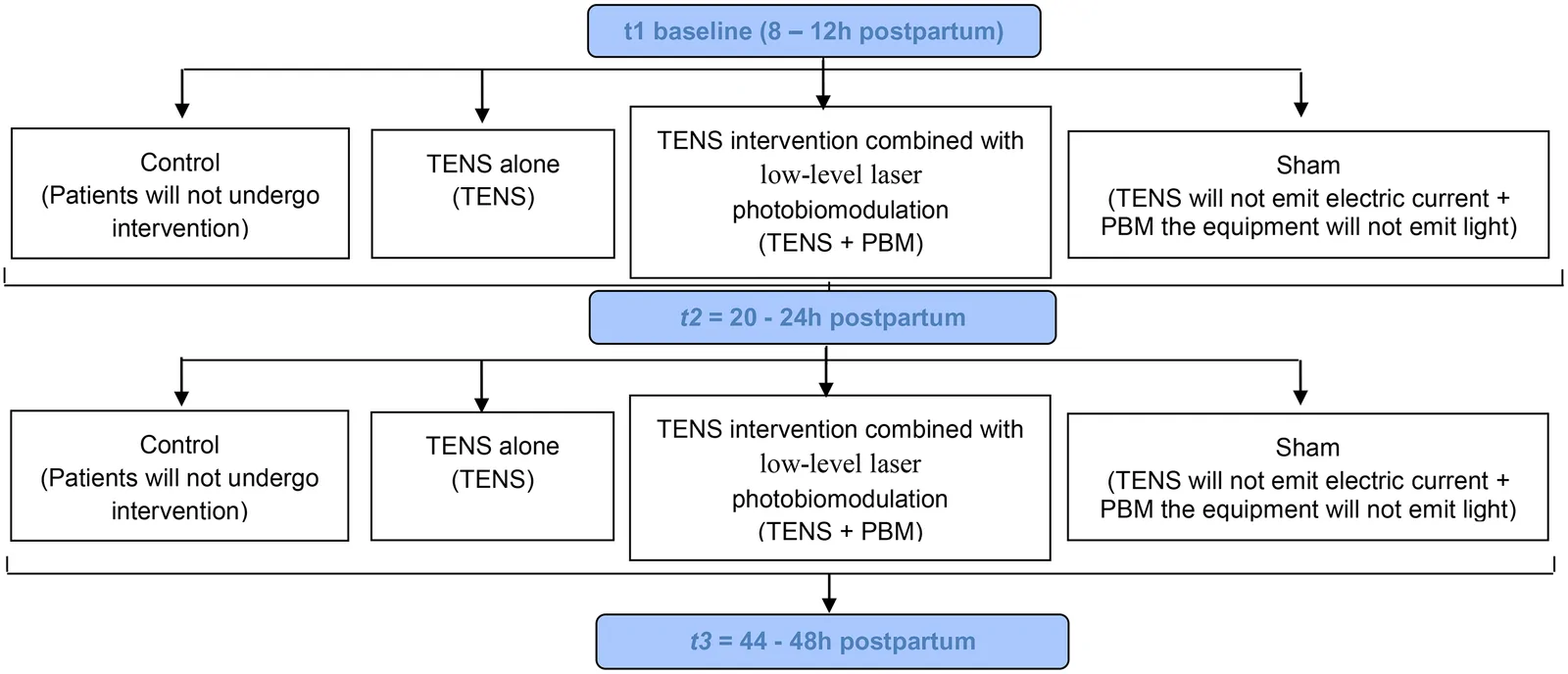
Study flowchart. Legend: t1: assessment 1; t2: assessment 2; t3: assessment 3; TENS: Transcutaneous Electrical Nerve Stimulation; PBM: Low-Level Laser Photobiomodulation.

All intervention sessions were conducted by a trained researcher, as well as the assessments, following the study of protocol of the Sena et al (2025) [12].

### TENS + Photobiomodulation Group

Participants were positioned in a supine neutral position and received simultaneous TENS and PBM. TENS was applied for 30 minutes using a frequency of 100 Hz and pulse duration of 75 μs, with intensity adjusted to patient tolerance. Two channels and four electrodes (5 × 3 cm) were placed above and below the surgical incision using conductive gel. Photobiomodulation was delivered using a 660 nm wavelength (red light) and continuo, applied perpendicularly in a point-by-point technique without direct contact with the incision (an average distance of 1 cm between the PBM device and the incision). Each point received 2 J (100 mW, 20 seconds per point), with 1 cm spacing between points.

### TENS Group

Participants received TENS only, using identical parameters and electrode placement as described above.

### Placebo Group

Participants received sham TENS (no current output) and inactive photobiomodulation (device without light emission), simulating the intervention procedures.

### Control Group

Participants received no therapeutic intervention and were only assessed at the predefined time points. All interventions and assessments were performed by trained researchers.

### Outcome measures

Assessments were conducted at three time points: baseline (t1: 8–12 h), post-intervention (t2: 20–24 h), and follow-up (t3: 44–48 h).

### Primary outcome: Pain

Pain was assessed in terms of intensity, pressure pain threshold, and tolerance. Pain intensity was measured using the Numeric Rating Scale (NRS), ranging from 0 (no pain) to 10 (worst imaginable pain). Pressure pain threshold and tolerance were assessed using a digital algometer applied perpendicularly at the midpoint between the umbilicus and the surgical incision (linea alba reference). Threshold was defined as the point at which pain was first perceived, and tolerance as the maximum pressure tolerated. Values were recorded in kgf/cm².

### Secondary outcomes

#### Functional independence.

Functional independence was assessed using an adapted Functional Independence Measure (FIM), covering self-care, mobility/transfers, and locomotion. Each item was scored from 1 (total dependence) to 7 (complete independence), with total scores ranging from 11 to 77.

Functional loss (%) was calculated as:


$$\begin{array}{c} \text{Functional loss}=\frac{\left(\text{initial score}-\text{final score}\right)}{\text{initial score}}\times \text{1}\text{0}\text{0} \end{array}$$

#### Functional capacity.

Functional capacity was assessed using the 2-minute walk test (2MWT), performed in a 30-meter corridor [13]. Two trials were conducted with a 30-minute rest interval, and the highest distance (meters) was recorded. Cardiorespiratory variables were measured before and immediately after the test. Perceived exertion was assessed using the Borg scale (6–20).

### Cardiorespiratory variables

The following variables were measured: systolic and diastolic blood pressure, heart rate, respiratory rate, and peripheral oxygen saturation (SpO_2_). Measurements were taken at baseline (t1) and, at t2 and t3, before, during, and immediately after the 2MWT.

### Ethical considerations

The study was approved by the Research Ethics Committee of the Federal University of Rio Grande do Norte (CAAE: 53263721.5.0000.5537) and registered at the Brazilian Registry of Clinical Trials (ReBEC: RBR-9pjd2n3) in July. All participants provided written informed consent prior to enrollment. The study adhered to national ethical regulations and ensured participant confidentiality and data protection.

The authors confirm that all ongoing and related trials for this intervention are registered.

### Statistical analysis

Data was analyzed using the Statistical Package for the Social Sciences (SPSS, version 20.0; IBM Corp., Armonk, NY, USA). Normality of data distribution was assessed using the Shapiro–Wilk test, and homogeneity of variances was evaluated with Levene’s test. Continuous variables were expressed as mean ± standard deviation (mean ± SD), while categorical variables were presented as absolute and relative frequencies (%). Baseline characteristics between experimental groups (age, gestational age, parity, education level, body mass index, and physical activity level) were compared using one-way ANOVA. In cases where assumptions of normality or homogeneity were violated, the non-parametric Kruskal–Wallis test was applied, followed by Tukey or Dunn post hoc tests, as appropriate. Within-group (intragroup) and between-group (intergroup) comparisons were conducted across three time points: t1 (8–12 hours postpartum), t2 (20–24 hours postpartum), and t3 (44–48 hours postpartum). To simultaneously assess the effects of the between-subject factor (Group: control, placebo, TENS combined with photobiomodulation, and TENS alone), the within-subject factor (Time: t1, t2, and t3), and their interaction effect (Group × Time), a two-way mixed repeated-measures ANOVA was performed.

When parametric assumptions were not met, the Friedman test was used for within-group comparisons, and the Kruskal–Wallis test for between-group comparisons, with Bonferroni correction applied for multiple comparisons. The level of statistical significance was set at α < 0.05. Effect sizes were also reported using partial eta squared (η²p), interpreted according to Cohen’s criteria (small < 0.06; medium = 0.06–0.14; large > 0.14), to enhance the clinical interpretation of the findings.

## Results

A total of 88 participants were screened according to the eligibility criteria and divided into 4 groups, according to the CONSORT flowchart. (Fig 2)

**Fig 2:**
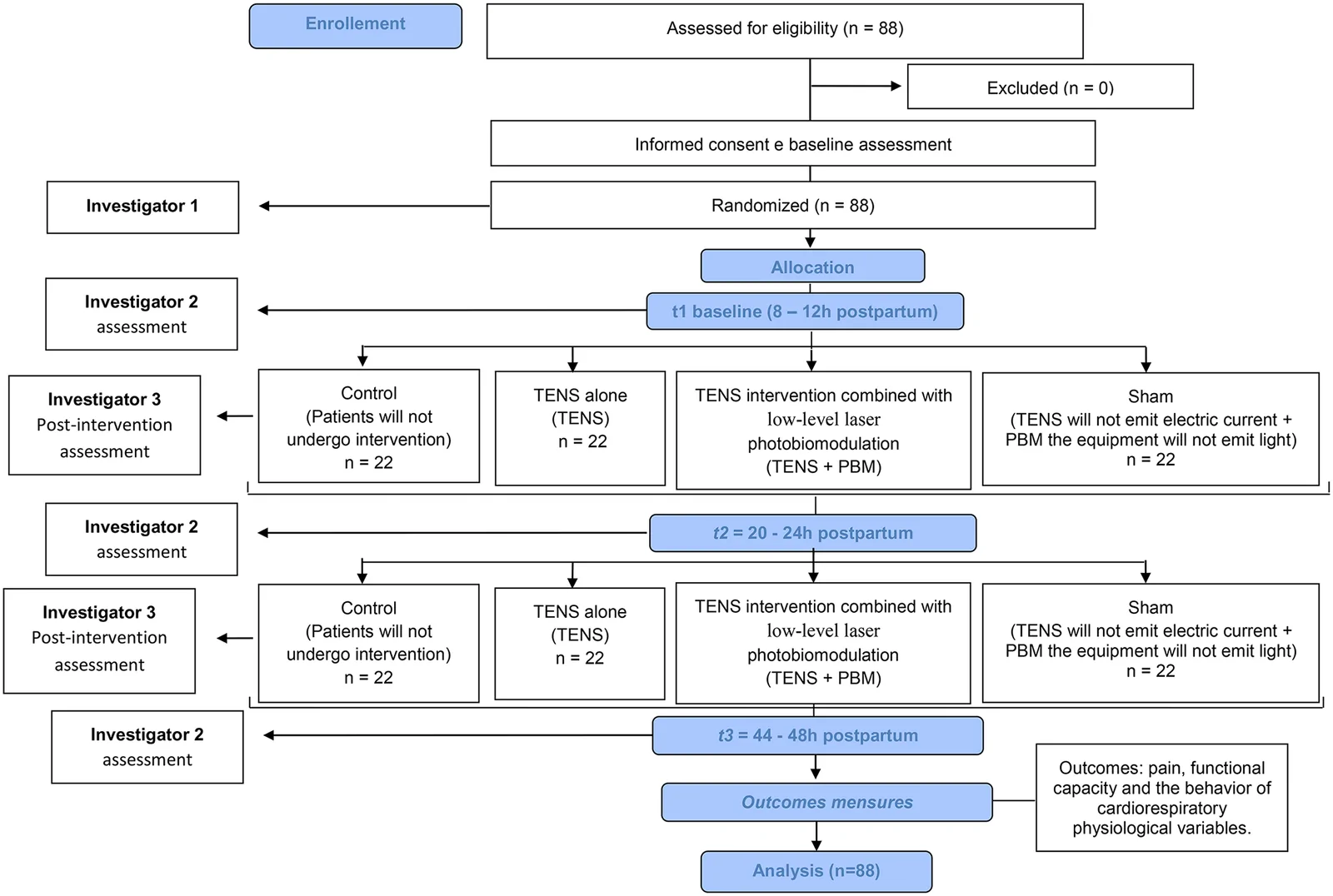
CONSORT Flowchart. Legend: t1 = assessment 1; t2 = assessment 2; t3 = assessment 3. n = sample size. TENS = Transcutaneous Electrical Nerve Stimulation; PBM = Low-Level Laser Photobiomodulation.

### Sociodemographic and clinical characteristics

Table 1 presents the characterization of participants across the four experimental groups, including means and standard deviations of the main sociodemographic and clinical variables. Mean age ranged from 27.3 ± 6.9 to 29.1 ± 6.3 years, indicating age-homogeneous samples. Mean gestational age was approximately 39 weeks across all groups, with minimal variation. The mean number of deliveries was similar between groups (ranging from 1.9 to 2.3), as was the average level of education, which ranged from 3.1 ± 1.2 to 4.0 ± 1.8 years of schooling. Body mass index (BMI) values ranged from 22.3 ± 6.5 to 24.5 ± 4.2 kg/m². Overall, the results indicate that the four groups were comparable, with no relevant differences observed in the analyzed variables.

**Table 1:** Sociodemographic and clinical characteristics of participants according to study groups (mean ± standard deviation) (n = 88).

Variables	G1	G2	G3	G4	p valor
**Age (years)**	27,3 ± 6,9	29,1 ± 6,3	28,5 ± 6,7	28,6 ± 7,4	0,8560
**Gestational age (weeks)**	39,1 ± 1,1	38,8 ± 1,3	38,1 ± 1,8	38,7 ± 1,7	0,1558
**Number of births**	1,9 ± 1,1	2,1 ± 1,1	2,3 ± 2,3	1,9 ± 0,8	0,6899
**Education level**	3,1 ± 1,2	4,0 ± 1,8	3,7 ± 1,6	3,3 ± 1,4	0,1952
**BMI**	24,5 ± 4,2	22,4 ± 4,4	22,3 ± 6,5	23,6 ± 4,5	0,5160
**IPAQ**					
**Sedentary**	6 (27,3%)	2 (9,1%)	9 (40,9%)	4 (19,0%)	0,29
**Irregularly Active**	7 (31,8%)	10 (45,5%)	8 (36,4%)	7 (33,3%)	
**Active**	0 (0,0%)	4 (18,2%)	0 (0,0%)	3 (14,3%)	
**Very Active**	9 (40,9%)	6 (27,3%)	5 (22,7%)	7 (33,3%)	

Data were presented as mean ± standard deviation, median [1st quartile – 3rd quartile], and relative frequency. A significant difference was considered for p < 0.05. No.: Number. BMI: Body Mass Index. IPAQ: International Physical Activity Questionnaire. %: Percent. G1: Control Group. G2: Placebo Group. G3: Intervention Group: Transcutaneous Electrical Nerve Stimulation combined with Low-Level Laser Photobiomodulation. G4: Intervention with Transcutaneous Electrical Nerve Stimulation.

### Baseline comparability and physical activity profile

None of the analyzed variables showed statistically significant differences between groups (all p > 0.05), indicating that the groups were homogeneous with respect to age, gestational age, parity, education level, body mass index (BMI), and physical activity level (IPAQ). Based on the International Physical Activity Questionnaire (IPAQ) classification, most participants across the four groups were categorized as either irregularly active or very active. The proportion of sedentary individuals ranged from 9.1% (Group 2) to 40.9% (Group 3). Notably, no participants in Groups 1 and 3 were classified as “active” (category 3), suggesting a lack of consistent engagement in moderate-intensity physical activity in these groups. Group 1 exhibited the highest proportion of very active individuals (40.9%), whereas Group 3 had the highest proportion of sedentary participants. Overall, these findings indicate variability in physical activity levels across groups; however, according to ANOVA results (p = 0.29), these differences did not reach statistical significance. This supports the interpretation that, despite percentage variations, overall physical activity behavior was comparable among the groups.

### Clinical and functional characteristics by Group

For statistical analysis, pain intensity assessed using the Numeric Rating Scale (NRS) was categorized as follows: 0 = no pain, 1 = mild pain, 2 = moderate pain, and 3 = severe pain. As shown in Table 2, intragroup analysis (t1 × t2 × t3) revealed statistically significant differences only for pain intensity (NRS 0–10), specifically in Groups 3 and 4, demonstrating a progressive and sustained reduction in pain scores over time (Fig 3). Similarly, Table 3 shows that intragroup analysis identified significant differences only for functional independence (FIM score), again in Groups 3 and 4, indicating improvement in functional independence over time (Fig 4). These findings suggest a clinically relevant improvement, potentially associated with the therapeutic effects of the interventions or with physiological adaptation during the postoperative recovery process.

**Table 2:** Temporal evolution of pain across groups, expressed as mean ± standard deviation, with statistical significance assessed using repeated-measures ANOVA or Friedman test.

Variable	Group	t1 (mean ± SD)	t2 (mean ± SD)	t3 (mean ± SD)	Intragroup p-value
**Pain (END 0–10)**	1	1.45 ± 1.18	1.82 ± 1.01	1.41 ± 0.96	0.412
	2	1.32 ± 0.78	1.41 ± 0.91	1.09 ± 0.87	0.288
	3	1.18 ± 0.91	1.82 ± 1.18	1.30 ± 0.86	**0.008**
	4	1.14 ± 0.83	1.59 ± 1.05	0.95 ± 1.05	**0.025**
**Algometry – Threshold (kgf/cm²)**	1	1.09 ± 0.64	1.22 ± 0.99	1.20 ± 1.24	0.768
	2	0.86 ± 0.60	0.73 ± 0.54	0.95 ± 0.65	0.226
	3	1.26 ± 0.74	1.21 ± 0.97	1.27 ± 0.81	0.669
	4	1.43 ± 1.16	1.18 ± 1.10	1.33 ± 1.10	0.503
**Algometry – Tolerance (kgf/cm²)**	1	1.72 ± 1.08	43.40 ± 195.80	1.64 ± 1.73	0.378
	2	1.23 ± 1.15	0.98 ± 0.78	1.29 ± 0.97	0.552
	3	2.04 ± 1.60	21.81 ± 91.25	1.71 ± 1.16	0.426
	4	1.88 ± 1.45	1.50 ± 1.35	1.59 ± 1.30	0.746

Data are presented as mean ± standard deviation. t1: assessment 1; t2: assessment 2; t3: assessment 3. kgf.cm2: Kilogram-force per square centimeter. G1: Control Group. G2: Placebo Group. G3: Intervention Group: Transcutaneous Electrical Nerve Stimulation combined with Low-Level Laser Photobiomodulation. G4: Intervention with Transcutaneous Electrical Nerve Stimulation. END: Numerical Pain Scale. Bold value indicates statistical significance (p < 0.05). ANOVA RM: Repeated measures ANOVA.

**Table 3:** Temporal evolution of clinical and functional variables by group, with means ± SD and statistical significance (repeated measures ANOVA/Friedman).

Variable	Group	t1 (mean ± SD)	t2 (mean ± SD)	t3 (mean ± SD)	Test	withingroup p-value	Clinical interpretation
**FIM (points)**	1	14.50 ± 3.10	15.00 ± 2.90	16.20 ± 3.40	ANOVA RM	0.061	Trend of functional improvement without statistical significance.
	2	15.10 ± 2.80	15.40 ± 3.00	16.70 ± 3.10	ANOVA RM	0.072	Slight increase in functional independence.
	3	14.90 ± 3.30	15.60 ± 3.20	17.10 ± 3.60	ANOVA RM	**0.034**	Significant improvement in functional independence.
	4	15.20 ± 3.00	16.10 ± 3.30	17.50 ± 3.40	ANOVA RM	**0.028**	Significant increase in functional autonomy.
**Heart rate (bpm)**	1	67.32 ± 11.47	81.90 ± 12.97	80.57 ± 13.97	ANOVA RM	0.204	Physiological variation with no statistical significance.
	2	71.05 ± 8.70	77.55 ± 12.43	89.38 ± 10.20	ANOVA RM	0.097	An upward trend without significance.
**RR (rpm)**	1	20.10 ± 2.30	21.30 ± 2.10	20.40 ± 2.20	ANOVA RM	0.648	Stable respiratory rate.
	2	19.80 ± 2.40	20.90 ± 2.20	20.30 ± 2.10	ANOVA RM	0.5710	Small fluctuation without statistical significance.
	3	20.00 ± 2.60	21.00 ± 2.30	20.20 ± 2.00	ANOVA RM	0.6240	Respiratory stability, within physiological limits.
	4	19.90 ± 2.50	21.10 ± 2.40	20.10 ± 2.10	ANOVA RM	0.6020	Respiratory rate preserved; no significant difference.
**O**_2_ **Saturation (%)**	1	96.20 ± 2.40	96.50 ± 2.00	97.10 ± 1.90	ANOVA RM	0.588	Adequate and constant oxygenation.
**SBP (mmHg)**	1	121.00 ± 15.20	122.40 ± 14.90	120.10 ± 13.80	ANOVA RM	0.8110	Systolic blood pressure stable; no significant changes.
	2	119.80 ± 14.60	121.20 ± 15.30	120.40 ± 14.10	ANOVA RM	0.7430	Values maintained between assessments.
	3	120.90 ± 15.10	122.00 ± 14.70	119.70 ± 13.90	ANOVA RM	0.7880	Systolic hemodynamic stability.
	4	122.30 ± 14.80	123.10 ± 15.20	121.00 ± 13.60	ANOVA RM	0.7020	Maintaining systolic blood pressure levels.
**DBP (mmHg)**	1	77.50 ± 10.30	76.80 ± 9.90	75.90 ± 10.10	ANOVA RM	0.6930	Diastolic control maintained between moments.
	2	76.90 ± 9.80	77.10 ± 10.40	76.50 ± 9.60	ANOVA RM	0.8210	No significant differences; diastolic stability.
	3	78.10 ± 10.50	77.40 ± 10.00	76.30 ± 9.80	ANOVA RM	0.7620	Diastolic hemodynamic stability.
	4	77.80 ± 10.20	77.00 ± 9.90	76.20 ± 10.00	ANOVA RM	0.7090	Diastolic pressure preserved; no statistical change.

Data are presented as mean ± standard deviation. t1: assessment 1; t2: assessment 2; t3: assessment 3. kgf.cm2: Kilogram-force per square centimeter. SBP: Systolic blood pressure. DBP: Diastolic blood pressure. mmHg: millimeters of mercury. HR: Heart rate. bpm: beats per minute. RR: Respiratory rate. bpm: breaths per minute. SpO2: Oxygen saturation. G1: Control group. G2: Placebo group. G3: Intervention group: Transcutaneous Electrical Nerve Stimulation combined with Low-Level Laser Photobiomodulation. G4: Intervention with Transcutaneous Electrical Nerve Stimulation. Bold values indicate statistical significance (p < 0.05). RM ANOVA: Repeated measures ANOVA.

**Fig 3:**
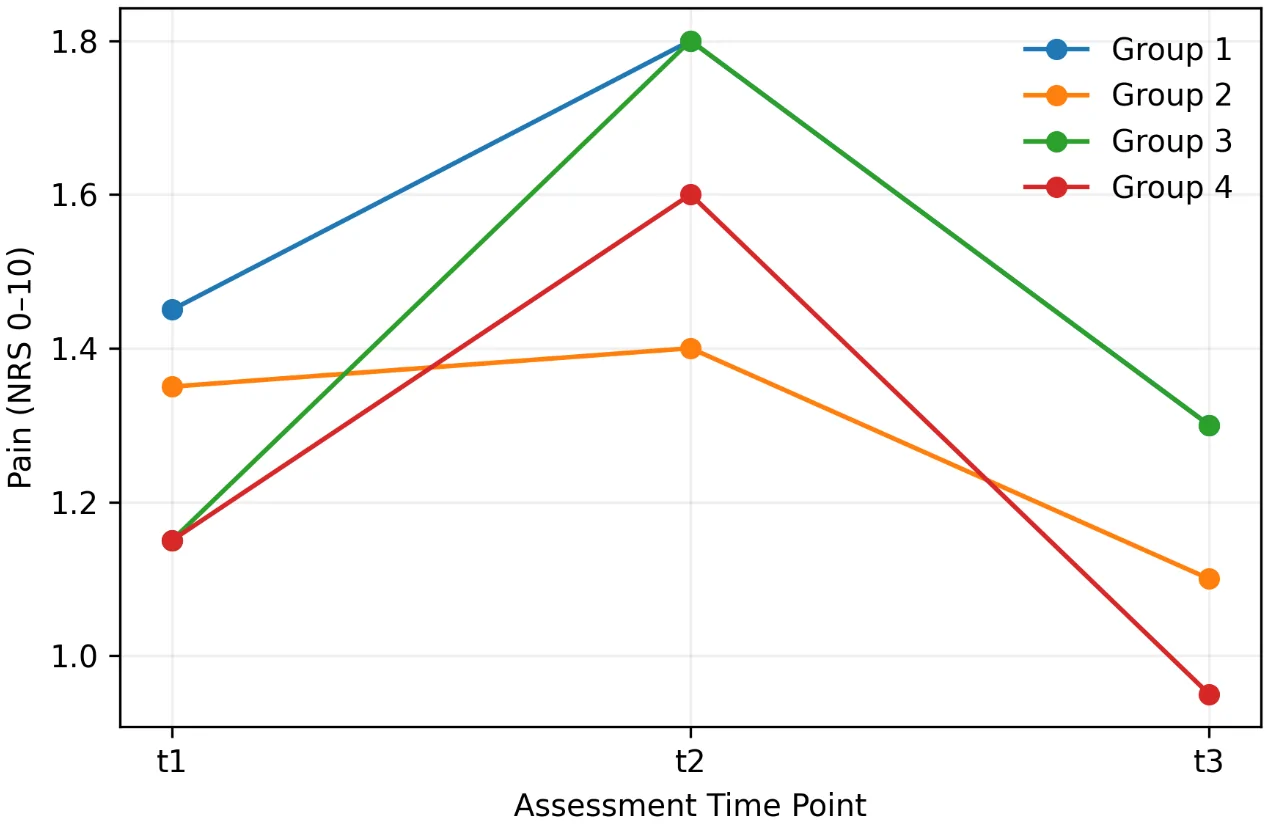
Intragroup evolution of pain at the three assessment times (t1, t2, and t3) for the four groups. G1: Control Group. G2: Placebo Group. G3: Intervention Group – Transcutaneous Electrical Nerve Stimulation combined with Low-Intensity Laser Photobiomodulation. G4: Intervention with Transcutaneous Electrical Nerve Stimulation. NDS: Numerical Pain Scale. Significant pain reduction in groups 3 and 4.

**Fig 4:**
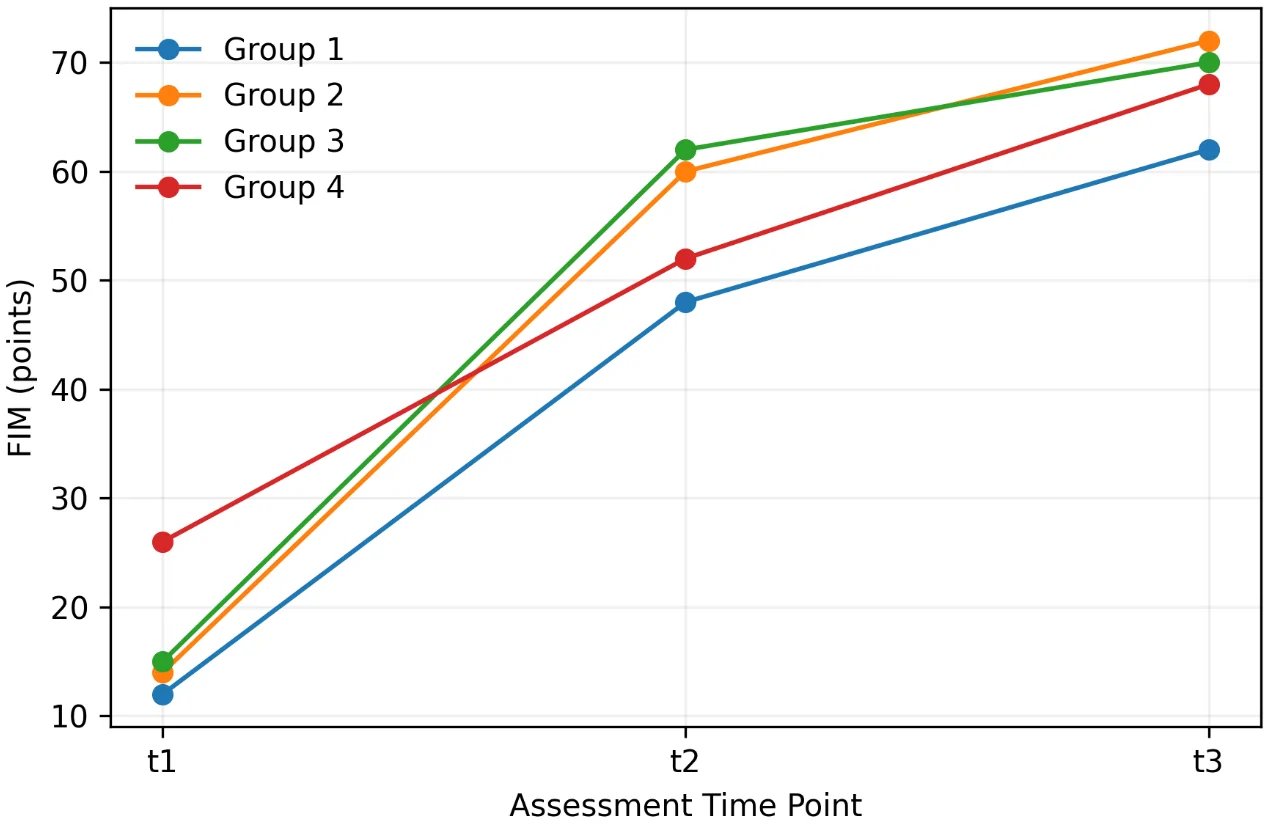
Intragroup evolution of the Functional Independence Measure (FIM) at the three assessment times (t1, t2, and t3) for the four groups. G1: Control Group. G2: Placebo Group. G3: Intervention Group – Transcutaneous Electrical Nerve Stimulation combined with Low-Intensity Laser Photobiomodulation. G4: Intervention with Transcutaneous Electrical Nerve Stimulation. FIM: Functional Independence Measure. Significant improvement in functional independence in groups 3 and 4.

Post hoc analyses were performed only for variables presenting significant overall effects in the mixed repeated-measures ANOVA. Pairwise comparisons with Bonferroni adjustment demonstrated a significant reduction in pain scores between t2 and t3 in Group 3 (adjusted p = 0.008) and Group 4 (adjusted p = 0.025), indicating progressive improvement in pain perception over time. In addition, functional independence (MIF) showed significant improvement between the initial and final assessments in Group 3 (adjusted p = 0.034) and Group 4 (adjusted p = 0.028), reflecting clinically relevant gains in functional performance throughout follow-up. No significant pairwise differences were observed for the remaining physiological or algometric variables (p > 0.05).

### Additional clinical outcomes

The remaining variables assessed, including hemodynamic parameters (systolic blood pressure [SBP], diastolic blood pressure [DBP], and heart rate [HR]), respiratory variables (respiratory rate [RR] and peripheral oxygen saturation [SpO_2_]) as presented in Table 3, and algometric measures (pain threshold and tolerance) in Table 2, did not show significant changes across time points. These findings indicate clinical stability and the absence of adverse physiological effects throughout the follow-up period. However, high inter-individual variability was observed in some measures, particularly in pain tolerance assessed by algometry. This variability may reflect heterogeneity in individual responses to the intervention and highlights the need for complementary analyses using more robust statistical approaches to better characterize intragroup response patterns (Table 2).

### Intergroup clinical and functional characteristics

As shown in Table 4, intergroup analysis demonstrated homogeneity among the four groups across all assessment time points (t1, t2, and t3), with no statistically significant differences observed in physiological, functional, or pain-related variables. These findings indicate that the groups had comparable baseline clinical conditions and exhibited similar trajectories over time, suggesting no relevant between-group differences in the behavior of the analyzed variables.

**Table 4:** Temporal evolution of clinical and functional variables between groups, expressed as mean ± standard deviation, with statistical significance assessed using repeated-measures ANOVA or Friedman test.

Variable	Moment	G1 (mean ± SD)	G2 (mean ± SD)	G3 (mean ± SD)	G4 (mean ± SD)	p-value
**Pain** **(END 0–10)**	t1	1.45 ± 1.18	1.32 ± 0.78	1.18 ± 0.91	1.14 ± 0.83	0.673
	t2	1.82 ± 1.01	1.41 ± 0.91	1.82 ± 1.18	1.59 ± 1.05	0.500
	t3	1.41 ± 0.96	1.09 ± 0.87	1.30 ± 0.86	0.95 ± 1.05	0.382
**Heart rate (bpm)**	t1	67.32 ± 11.47	71.05 ± 8.70	71.00 ± 15.81	71.36 ± 9.44	0.623
	t2	81.90 ± 12.97	77.55 ± 12.43	80.05 ± 11.79	85.30 ± 16.65	0.328
	t3	80.57 ± 13.97	89.38 ± 10.20	84.21 ± 13.01	91.05 ± 19.27	0.087
**Algometry** **Threshold** **(kgf/cm²)**	t1	1.09 ± 0.64	0.86 ± 0.60	1.26 ± 0.74	1.43 ± 1.16	0.128
	t2	1.22 ± 0.99	0.73 ± 0.54	1.21 ± 0.97	1.18 ± 1.10	0.232
	t3	1.20 ± 1.24	0.95 ± 0.65	1.27 ± 0.81	1.33 ± 1.10	0.597
**Algometrics Tolerance** **(kgf/cm²)**	t1	1.72 ± 1.08	1.23 ± 1.15	2.04 ± 1.60	1.88 ± 1.45	0.216
	t2	43.4 ± 195.8	0.98 ± 0.78	21.81 ± 91.25	1.50 ± 1.35	0.528
	t3	1.64 ± 1.73	1.29 ± 0.97	1.71 ± 1.16	1.59 ± 1.30	0.746
**2MWT (m)**	t2	63.50	50.33	48.32	61.10	0.1254
	t3	76.75	63.86	65.21	72.20	0.2481

Data are presented as mean ± standard deviation. t1: assessment 1; t2: assessment 2; t3: assessment 3. kgf.cm2: Kilogram-force per square centimeter. 2MWT: Two-Minute Walk Test. m: meter. mmHg: millimeters of mercury. HR: Heart Rate. bpm: beats per minute. RR: Respiratory Rate. bpm: Respiratory Incursions per Minute. SpO2: Oxygen Saturation. G1: Control Group. G2: Placebo Group. G3: Intervention Group – Transcutaneous Electrical Nerve Stimulation combined with Low-Level Laser Photobiomodulation. G4: Intervention Group – Transcutaneous Electrical Nerve Stimulation. NRS: Numerical Pain Scale.

However, intragroup variability was observed in certain parameters, particularly in the algometric measures, which may reflect heterogeneous individual responses or the influence of personal and contextual characteristics of the participants. In summary, the results corroborate the consistency and comparability between the groups, ensuring that the changes identified in the intragroup analyses represent real changes over time within each group, and not pre-existing differences between them. The intragroup analysis of the Two-Minute Walk Test (2MWT) revealed a significant improvement in performance between time 2 (t2) and time 3 (t3) in all groups evaluated. Group 1 showed an average increase from 63.50 to 76.75 meters (paired t, p = 0.0022), while group 2 improved from 50.33 to 63.86 meters (Wilcoxon, p = 0.0022). Similarly, group 3 showed an increase from 48.32 to 65.21 meters (Wilcoxon, p = 0.0006) and group 4 from 61.10 to 72.20 meters (paired t, p = 0.0053). These results demonstrate significant functional gains in physical performance over the period, regardless of the intervention group, indicating a consistent improvement in gait capacity between t2 and t3.

In the intergroup analysis, comparing the four groups with each other, no statistically significant differences were observed in the distances covered in the Two-Minute Walk Test (2MWT) at any of the times evaluated. At time 2 (t2), the Kruskal-Walli’s test indicated no significant difference between the groups (p = 0.1254), while at time 3 (t3), the one-way ANOVA test confirmed the same pattern (p = 0.2481). These findings suggest that, despite the previously observed intragroup functional improvement, the groups maintained equivalent performances with each other at the two evaluation times.

Fig 5 represents the intergroup comparison of pain scores (0–10) assessed using the NDS at the three evaluation times (t1, t2, and t3). Each group (1–4) is illustrated by a distinct color, and the bars indicate the mean pain score, while the error lines represent the standard deviation, highlighting the internal variability of each group.

**Fig 5:**
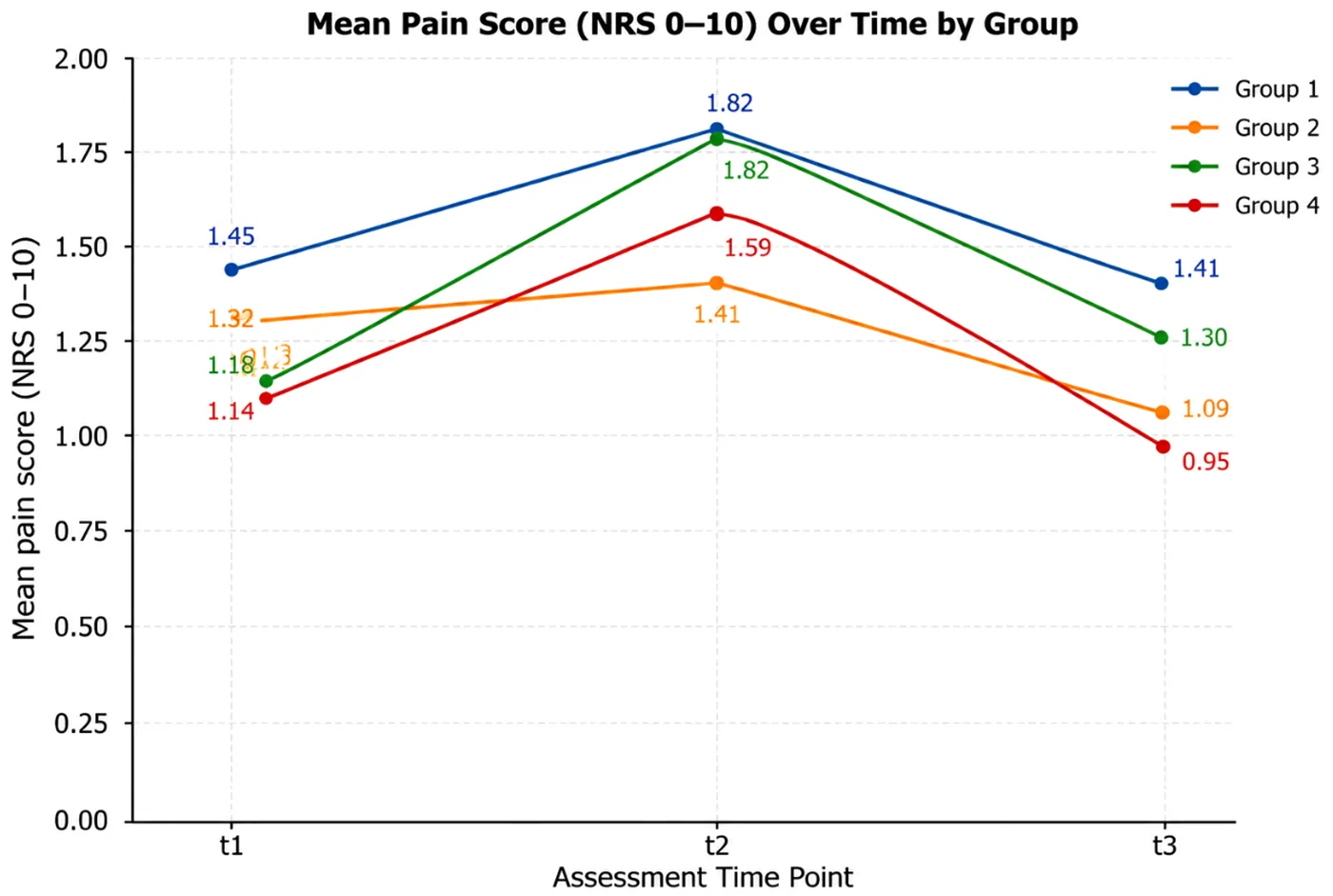
Comparison of pain intensity between groups at different evaluation times. G1: Control Group. G2: Placebo Group. G3: Intervention Group: Transcutaneous Electrical Nerve Stimulation combined with Low-Level Laser Photobiomodulation. G4: Intervention with Transcutaneous Electrical Nerve Stimulation. NDS: Numerical Pain Scale. Significant improvement in Functional Independence in groups 3 and 4.

Visual analysis shows that, at all evaluated times, the groups presented similar average pain values, without significant differences between them, which corroborates the results obtained by the one-way ANOVA test, which did not identify statistical significance between the groups (p > 0.05). Between time points t1 and t3, a general trend of reduction in average pain scores is observed, especially in groups 3 and 4, although this decrease was not significant in comparison with the groups. These findings indicate a parallel clinical evolution and homogeneity between the groups, reinforcing that the variations detected in the intragroup analyses reflect individual improvements within each group, and not structural differences between them.

To investigate the interaction between the between-subjects factor (group: 1, 2, 3 and 4) and the within-subjects factor (time: t1, t2 and t3), a two-way mixed ANOVA was applied. This model allows us to identify whether the changes observed over time (main effect of time) occurred similarly or differently between the groups (group × time interaction effect). The results indicated no statistically significant interaction between group and time (p > 0.05) for all variables evaluated, including physiological parameters (SBP, DBP, HR, RR, and SpO_2_), algometric parameters (pain threshold and tolerance), and functional parameters (FIM). This finding suggests that the groups evolved in parallel throughout the evaluations, without relevant differences in the temporal response pattern between them.

There was a significant main effect of time for the pain variable (p < 0.05), reflecting a reduction in pain scores between T2 and T3, especially in groups 3 and 4. For the FIM variable, a significant increase in scores was observed over time (p < 0.001) in all groups, indicating a progressive improvement in functional independence, but without an interaction effect (p > 0.05), demonstrating that this improvement occurred similarly across groups.

### Group and time effect

The results of the mixed ANOVA (group x time) confirm that the changes observed throughout the assessments occurred consistently among the groups, with no indication that any group showed a different response to the interventions. Thus, the findings reinforce the homogeneity of the experimental conditions and the overall effectiveness of the interventions in improving function and reducing pain throughout the follow-up. The two-factor mixed analysis of variance (group x time) indicated no significant interaction between group and time for all physiological, functional, and algometric variables evaluated (p > 0.05). This finding suggests that the changes observed throughout the three assessments (t1, t2, and t3) occurred similarly among the four groups, with no difference in the temporal response pattern among them.

The main group effect also showed no statistical significance (F₍3,𝑛₎ < 2.0; p > 0.05; η²ₚ < 0.05), indicating homogeneity between the groups in all outcomes analyzed, including physiological variables, systolic blood pressure (SBP), diastolic blood pressure (DBP), heart rate (HR), respiratory rate (RR), and peripheral oxygen saturation (SpO_2_), as well as measures of functional independence (FII) and pain threshold and tolerance. On the other hand, a significant main time effect was observed for the pain variable (0–10) (F₍2,₎ ≈ 4.8; p = 0.012; η²ₚ = 0.11), demonstrating a progressive reduction in mean pain scores between T2 and T3, mainly in groups 3 and 4. This result reflects a noticeable improvement in pain symptoms, possibly associated with physiological adaptation or the positive impact of the therapeutic intervention.

The Functional Independence Measure (FIM) variable showed a highly significant time effect (F₍2,₎ ≈ 9.6; p < 0.001; η²ₚ = 0.21), indicating a consistent increase in mean scores throughout the assessments in all groups, which demonstrates an overall improvement in the functional independence of the participants. However, the group x time interaction effect for MIF did not reach significance (p > 0.05), indicating that the pattern of evolution was similar between the groups. In general, the results confirm that time exerted a significant influence on the clinical improvement of the participants, reflected mainly in the reduction of pain and functional improvement, while the groups presented parallel trajectories of evolution, without relevant statistical differences between them.

## Discussion

The findings of this study demonstrate that, across the three assessment time points (t1, t2, and t3), participants showed significant improvements in clinical and functional outcomes, regardless of group allocation. These results are consistent with existing evidence highlighting the natural course of recovery following cesarean delivery, as well as the beneficial effects of early physiotherapeutic interventions. Previous studies indicate that, during the first postoperative weeks, there is a progressive reduction in pain, edema, and local sensitivity, accompanied by functional improvement driven by tissue remodeling and the gradual restoration of abdominal and lumbopelvic mobility [14,15].

Randomized controlled trials have also shown that the early introduction of physiotherapeutic strategies, including mobilization, proprioceptive stimulation, and electrotherapy, can accelerate functional recovery and enhance maternal autonomy, even when the isolated effects of specific techniques do not differ significantly between groups [13]. Furthermore, systematic reviews investigating transcutaneous electrical nerve stimulation (TENS) in postoperative pain management report that, although TENS may reduce pain intensity and analgesic consumption, the overall recovery trajectory and continuous physiotherapeutic care remain key determinants of clinical and functional improvement [16]. Therefore, the present findings align with contemporary literature, suggesting that temporal progression combined with physiotherapeutic care plays a predominant role in post-cesarean recovery, regardless of the specific combination of techniques applied.

The consistent reduction in pain (pain variable) and improvement in functional independence (FIM variable), observed as a significant time effect, further support the concept of progressive recovery following cesarean delivery, independent of the specific intervention. A important finding of the present study was the absence of significant differences between the intervention groups. However, both the TENS + PBM and TENS alone groups demonstrated significant pain reduction over time, suggesting that these interventions may contribute to post-cesarean pain management.

Evidence indicates that, in the early postoperative period, pain reduction is closely linked to physiological tissue healing and functional reorganization of the trunk and abdominal wall, directly influencing maternal functional capacity [14,15]. Additionally, the literature supports that early physiotherapy protocols, including light mobilization and sensorimotor stimulation, contribute to faster recovery of functional independence and reduction of limitations in activities of daily living [13]. Recent systematic reviews on TENS and postoperative rehabilitation also demonstrate significant improvements in pain and functional outcomes over time, irrespective of whether interventions are applied in isolation or combination, suggesting a dominant effect of the “recovery time” factor and the overall rehabilitation approach [16]. Thus, the present findings corroborate the growing body of evidence indicating that pain reduction and functional gains are interdependent processes influenced by physiological repair mechanisms, therapeutic interventions, and the natural course of postoperative recovery.

The stability of physiological variables (SBP, DBP, HR, RR, and SpO_2_), as well as functional capacity and algometric measures (pain threshold and tolerance), reinforces that the observed improvements were not associated with adverse hemodynamic or physiological changes, but rather with favorable adaptive and functional adjustments. Similar findings have been reported in studies evaluating TENS and physiotherapy in postpartum women following cesarean delivery, demonstrating reductions in pain and improvements in function without negative effects on cardiovascular or respiratory responses [14,13]. These results support the physiological safety of TENS, which primarily acts through peripheral and central neuromodulatory mechanisms—such as activation of A-β fibers and endogenous opioid release—without significantly affecting systemic hemodynamics [16]. Moreover, recent reviews emphasize that effective pain control and early mobilization contribute to cardiorespiratory homeostasis, reduction of thromboembolic complications, and optimization of functional recovery in postpartum women [13,17].

In this context, the stability of physiological parameters alongside increased pain threshold and tolerance observed in the present study further supports current evidence that safe and progressive physiotherapeutic interventions can promote functional adaptation and effective analgesia without inducing hemodynamic or respiratory imbalance.

### Limitations

The original sample size calculation was based on expected between-group differences and did not explicitly account for the potential group × time interaction inherent to the repeated-measures design. Future studies should consider sample size methods specifically tailored for mixed-model or repeated-measures analyses.

Another relevant factor for this study is that a fifth group receiving PBM intervention alone could have provided more robust comparative data with the other groups. However, this research was conducted based on a previously established clinical trial protocol, and therefore we followed the methodology as described in the protocol, which did not include a PBM only group.

## Conclusion

This randomized clinical trial demonstrated that the absence of a significant group × time interaction indicates that the different experimental protocols or conditions produced similar effects on the analyzed outcomes, reflecting parallel trajectories across groups. A significant main effect of time was observed for pain and functional independence (FIM), suggesting that the intervention process, monitoring, or follow-up contributed to a consistent reduction in pain and improvement in functional autonomy among participants. In contrast, the stability of physiological variables (SBP, DBP, HR, RR, and SpO_2_), as well as functional capacity and algometric measures (pain threshold and tolerance), indicates that the observed changes were not associated with adverse hemodynamic or physiological alterations, but rather with favorable adaptive and functional adjustments. Overall, these findings suggest that time was a determining factor in the improvements observed across all groups, with homogeneous clinical responses, supporting the consistency and effectiveness of the implemented program. These results reinforce the importance of continuity and adherence to therapeutic strategies in promoting functional gains and pain control in similar clinical populations.

## Supporting information

S1 File Check List CONSORT. (DOCX)

S2 File Trial Protocol. (DOC)
