# Supplementary material for: Combined transcutaneous electrical nerve stimulation and low-level laser photobiomodulation for postoperative pain after cesarean section: A randomized controlled trial
Source: PLoS One. 2026 Oct 22;21(10):e0360761. doi: 10.1371/journal.pone.0360761 (PMC13690222; doi:10.1371/journal.pone.0360761)
Supplement: Supplementary file 2 [file pone.0360761.s002.doc]

**Fig 2.** CONSORT Flow chart.

**Allocation**

**Enrollement**

***t2* = 20 - 24h postpartum**

**Analysis (n=88)**

***t3* = 44 - 48h postpartum**

**t1 baseline (8 – 12h postpartum)**

***Outcomes mensures***

Assessed for eligibility (n = 88)

Excluded (n = 0)

Control

(Patients will not undergo intervention)

n = 22

Randomized (n = 88)

TENS alone

(TENS)

n = 22

Sham

(TENS will not emit electric current + PBM the equipment will not emit light)

n = 22

Informed consent e baseline assessment

Control

(Patients will not undergo intervention)

n = 22

TENS alone

(TENS)

n = 22

Sham

(TENS will not emit electric current + PBM the equipment will not emit light)

n = 22

Outcomes: pain, functional capacity and the behavior of cardiorespiratory physiological variables.

**Investigator 1**

**Investigator 2**

assessment

TENS intervention combined with low-level laser photobiomodulation

(TENS + PBM)

n = 22

**Investigator 3**

Post-intervention assessment

**Investigator 2**

assessment

TENS intervention combined with low-level laser photobiomodulation

(TENS + PBM)

(TENS + LLLT)

n = 22

**Investigator 3**

Post-intervention assessment

**Investigator 2**

assessment

**t1:** assessment 1; **t2:** assessment 2; **t3:** assessment 3; **TENS:** Transcutaneous Electrical Nerve Stimulation; **PBM:** Low-level laser photobiomodulation.
